# Trajectory patterns for continuous metabolic syndrome score in childhood and the cardiovascular risk in adolescence

**DOI:** 10.1038/s41598-021-01566-y

**Published:** 2021-11-19

**Authors:** Eun Jeong Choi, Hye Ah Lee, Bomi Park, Bohyun Park, Hae Soon Kim, Young Sun Hong, Hyesook Park

**Affiliations:** 1 Department of Preventive Medicine, College of Medicine, Ewha Womans University, Seoul, Republic of Korea; 2 Clinical Trial Center, Ewha Womans University Medical Center, Seoul, Republic of Korea; 3 Department of Preventive Medicine, College of Medicine, Chung-Ang University, Seoul, Republic of Korea; 4 National Cancer Control Institute, National Cancer Center, Goyang, Republic of Korea; 5 Department of Pediatrics, College of Medicine, Ewha Womans University, Seoul, Republic of Korea; 6 Department of Internal Medicine, College of Medicine, Ewha Womans University, Seoul, Republic of Korea; 7 Graduate Program in System Health Science and Engineering, Ewha Womans University, Seoul, Republic of Korea

**Keywords:** Epidemiology, Cardiovascular diseases

## Abstract

We explored the association between the trajectory of the continuous metabolic syndrome score (cMetS) in childhood with high-sensitivity C-reactive protein (hs-CRP) and carotid intima-media thickness (CIMT), which are known to increase cardiovascular disease risk in adolescence. The trajectory of cMetS in childhood (from 3 to 12 years of age) was identified in 833 children who participated in the Ewha Birth and Growth Study. The associations between cMetS and hs-CRP and CIMT were analyzed in 204 out of 833 children who participated in the follow-up at 13–15 years of age and measured hs-CRP and CIMT. Among the 833 children, three groups were classified: cMetS maintained at a low level (n = 198, 23.77%), middle level (n = 530, 63.63%), and at high levels (n = 105, 12.61%). The group with a stable-high cMetS trajectory showed significantly higher hs-CRP levels, and the statistical significance was maintained after adjusting for covariates. This study found that a consistently high cMetS in childhood was significantly associated with higher hs-CRP levels in adolescents, suggesting that it is necessary to intervene in metabolic risk factors early in life to reduce the risk of cardiovascular disease later in life.

## Introduction

Cardiovascular disease is the number one cause of death worldwide, with the World Health Organization reporting that approximately 17.9 million people died from cardiovascular disease in 2016, which accounted for 31% of all deaths worldwide^1^. In addition, heart disease and cerebrovascular disease occupy the second and fourth places, respectively, among the top 10 causes of death in the 2019 Cause of Death statistics in South Korea^2^.

An increase in high-sensitivity C-reactive protein (hs-CRP), a nonspecific inflammatory indicator, was reported to be associated with a higher risk of cardiovascular disease^3–5^. In addition, it was reported that hs-CRP levels in adolescents diagnosed with metabolic syndrome (MetS) were higher than those in undiagnosed adolescents, and there was a high correlation between the severity of MetS and hs-CRP levels in children and adolescents^6^. An increase in carotid intima-media thickness (CIMT), a marker of preclinical atherosclerosis, has also been reported to be associated with metabolic components, including hypertension, dyslipidemia, and diabetes, and MetS^7,8^. According to the Muscatine Study conducted in the United States in 2011, high body mass index (BMI), cholesterol, triglyceride (TG), and blood pressure (BP) observed at 8–18 years of age were significantly associated with increased CIMT at 33–42 years of age^9^. A study conducted in Korea also reported that obese children and adolescents had a significantly thicker average CIMT than the normal-weight group^10^. Other studies in children and adolescents have also reported that obesity contributes to increased CIMT^11^.

Early intervention in childhood and adolescence for metabolic components such as obesity, hypertension, and dyslipidemia seems to be important because these poor health conditions tend to persist over time. In the Malaysian Health and Adolescents Longitudinal Research Team (MyHeART) study, adolescents aged 13–17 years were stratified into low-, medium-, and high-risk groups according to cardiovascular disease risk factors, and more than 12% of children who were categorized as the high-risk group remained in the high-risk group at 15–17 years of age^12^. Although this study evaluated the trajectory of poor metabolic health, the evaluation period was relatively short.

Thus, based on the Ewha Birth and Growth Study, we aimed to identify the trajectory patterns of continuous metabolic syndrome score (cMetS) in children from 3 to 12 years of age, and to evaluate the association between the cMetS trajectories in childhood and hs-CRP and CIMT in adolescence.

## Materials and methods

### Study subjects

The Ewha Birth and Growth Study was conducted with women who visited Ewha Womans University Mokdong Hospital for prenatal examination at 24 to 28 weeks of pregnancy from 2001 to 2006 and agreed to participate in the cohort study. Since 2005, follow-up evaluations of their children have been conducted at the ages of 3, 5, and 7 years of age and annually thereafter. Details of the cohort have been previously reported^13^. Anthropometric measurements, blood and urine sample collection, and questionnaire completion were performed at each follow-up. To analyze the cMetS trajectory, this study included 833 subjects who participated at the follow-ups at least once between 3 and 12 years of age and measured the metabolic indicators constituting cMetS. In addition, 204 out of 833 subjects who participated at the follow-up at 13 − 15 years of age and had no missing values were included in the second analysis to explore the association between cMetS trajectory and hs-CRP and CIMT.

The subjects who were lost to follow-up were more likely to have had a low birth weight or preterm birth than the study subjects. However, there was no difference in sex distribution or maternal characteristics (Supplementary Table I).

### Indicators of metabolic health

The cMetS at each follow-up age was calculated using BMI, fasting blood glucose, TG, mean arterial pressure (MAP), and high-density lipoprotein-cholesterol (HDL-c) to determine the risk of MetS in children. Anthropometric measurements were performed by trained researchers. BMI (kg/m^2^) was calculated from the height and weight measured with the subjects wearing no shoes and light clothing using a stadiometer and a calibrated scale (DS-102, Dong Sahn Jenix, Co., Ltd, Seoul, Korea). BMI was calculated as body weight (kg) divided by height squared (m^2^). Blood chemistry tests were performed for fasting blood glucose, TG, and HDL-c levels, as well as a complete blood count. BP was measured twice using an automatic device (Dinamap Procure 200, GE, Milwaukee, WI) with an appropriate cuff size after the participants had been rested for five min. Two measurements, taken within 5 min of each other, were averaged. MAP was calculated using the following formula: MAP (mmHg) = diastolic BP + [(systolic BP—diastolic BP)/3]. To calculate cMetS, each metabolic indicator was standardized by age group with consideration of sex using the Z-score method. Z-scores of BMI, fasting blood glucose, TG, MAP, and HDL-c multiplied by -1 were summed to calculate cMetS^14,15^.

### Outcomes

As outcomes, hs-CRP and CIMT measured at 13 to 15 years of age were considered. Blood hs-CRP levels were measured using a particle-enhanced immune turbidimetric assay (Cobas 8000 C702 analyzer; Roche, Mannheim, Germany). The limit of detection (LOD) for hs-CRP is 0.15 mg/dL, and the value equivalent to the LOD/√2 was assigned to the values lower than LOD. The hs-CRP level was measured by the Seegene Medical Foundation (Seoul, Korea), and the coefficient of variance in terms of quality assurance/quality control was less than 10% in all measurements. hs-CRP did not satisfy the normal distribution despite log-transformed data, so subjects were divided according to 0.3 mg/dL of hs-CRP^16^.

CIMT was measured from the left and right carotid arteries to the first decimal place by an ultrasound expert using carotid ultrasound (Vivid E9; GE Vingmed Ultrasound AS, Horten, Norway). Additionally, the average values of the left and right measurements were calculated and used for the analysis.

### Covariates

Through a review of the literature, sex, age, monthly household income, frequency of vigorous-intensity physical activity, disease history of parents, and exposure to secondhand smoke were selected as confounding variables^17–19^. The information for covariates was collected from the questionnaires. Monthly household income was divided into three categories (less than 3 million won, 3–5 million won, and 5 million won or more) according to the frequency distribution. The vigorous-intensity physical activity per week was categorized as 1–2 times per week, 3–4 times per week, and ≥ 5 times per week. The disease history of parents was considered present if any cardiovascular disease, dyslipidemia, hypertension, or diabetes were present. We did not use pubertal maturation as a covariate in our study because only 4 of 96 boys and 2 of 108 girls were prepubertal according to the responses to a questionnaire^20^.

### Statistical analysis

For descriptive statistics of the variables, the mean and standard deviation were calculated to two decimal places for normally distributed continuous variables. Non-normally distributed continuous variables are shown as median and interquartile range (IQR) to two decimal places. Categorical variables are presented as frequencies with percentages.

In order to categorize participants into different subgroups according to the patterns of change in cMetS, trajectory analysis was performed using PROC TRAJ in the SAS program. The most appropriate final model was selected based on the group distribution, Akaike information criterion (AIC), and Bayesian information criterion (BIC)^21,22^. PROC MIXED was used to calculate the intra-class correlation coefficient (ICC) of metabolic components repeatedly measured between 3 and 12 years of age. Additionally, a correlation analysis was performed on the data measured at two adjacent time points.

An analysis of variance was conducted on the mean difference of CIMT in adolescence by groups categorized based on the cMetS trajectory. Analysis of covariance was conducted after adjusting for covariates. The association between elevated hs-CRP levels and cMetS trajectory group was evaluated using uni- and multivariate logistic models. Model 1 was adjusted for sex, while Model 2 was also adjusted for monthly household income, frequency of vigorous-intensity physical activity, disease history of parents, and exposure to secondhand smoke. A sensitivity analysis based on previous studies^23,24^ was conducted, excluding subjects with hs-CRP > 1 mg/dL. We also assessed gender differences in the associations of cMetS trajectory group with hs-CRP level and CIMT. All statistical analyses were performed using SAS software (version 9.4; SAS Institute, Cary, NC, USA), and statistical significance in the two-tailed test was determined as a *p*-value < 0.05.

### Ethical approval and informed consent

Written informed consent for the study was obtained from all subjects, parents, or guardians for participation in the study, and the study protocol was approved by the Institutional Review Board of Ewha Womans University Seoul Hospital (IRB number: SEUMC 2019–04-034). All methods of this study were performed in accordance with the relevant guidelines and regulations. Furthermore, this study was performed in compliance with the principles set forth in the Declaration of Helsinki.

## Results

### Descriptive summary of the cMetS and cMetS components

BMI gradually increased with age from 15.45 ± 1.37 kg/m^2^ at the age of 3 to 15.97 ± 2.12 kg/m^2^ at the age of 7 and 19.82 ± 3.10 kg/m^2^ at the age of 12 years of age. MAP also gradually increased with age, being 71.36 ± 8.36 mmHg at 3, 72.47 ± 6.88 mmHg at 7, and 82.19 ± 8.83 mmHg at 12 years of age. The mean standardized value of cMetS was 0.00, at each time point, and the standard deviation range was 2.64 to 2.88, showing a similar distribution (Table 1).

**Table 1:** Descriptive summary of metabolic syndrome components and cMetS by follow-up time points.

		Follow-up time points								
		3 years (n = 447)	5 years (n = 375)	7 years (n = 354)	8 years (n = 378)	9 years (n = 394)	10 years (n = 233)	11 years (n = 269)	12 years (n = 227)	13–15 years (n = 204)
Gender ratio (Boys/Girls)		0.97	1.05	0.95	1.09	1.01	1.10	0.96	1.01	0.89
BMI (kg/m^2^)		15.45 ± 1.37	15.70 ± 1.61	15.97 ± 2.12	16.82 ± 2.59	17.52 ± 2.61	18.01 ± 2.90	18.91 ± 3.06	19.82 ± 3.10	20.39 ± 3.12
MAP (mmHg)		71.36 ± 8.36	72.51 ± 6.54	72.47 ± 6.88	75.45 ± 7.69	77.38 ± 7.25	83.17 ± 9.03	84.13 ± 8.53	82.19 ± 8.83	80.98 ± 8.47
TG (mg/dL)^a^		56.00 (41.00 ~ 79.00)	46.00 (34.00 ~ 67.00)	50.50 (35.00 ~ 77.00)	56.00 (40.00 ~ 70.00)	61.50 (43.00 ~ 88.00)	61.00 (48.00 ~ 80.00)	68.00 (52.00 ~ 93.00)	71.00 (51.00 ~ 93.00)	62.00 (49.00 ~ 84.00)
FBG (mg/dL)		79.61 ± 8.63	78.23 ± 9.24	80.48 ± 7.14	81.19 ± 7.06	82.48 ± 7.51	93.09 ± 6.45	93.87 ± 6.26	92.65 ± 6.26	92.92 ± 6.55
HDL-c (mg/dL)		54.60 ± 10.81	60.23 ± 11.86	61.30 ± 11.67	61.83 ± 11.53	60.81 ± 11.34	57.59 ± 11.44	55.41 ± 11.49	53.89 ± 10.89	51.07 ± 9.70
cMetS		0.00 ± 2.64	0.00 ± 2.64	0.00 ± 3.02	0.00 ± 2.86	0.00 ± 2.87	0.00 ± 2.88	0.00 ± 2.83	0.00 ± 3.01	0.00 ± 2.83

SD, standard deviation; BMI, body mass index; MAP, mean arterial pressure; TG, triglyceride; FBG, fasting blood glucose; HDL-c, high-density lipoprotein-cholesterol; cMetS, continuous metabolic syndrome score.

Mets components are presented as means and standard deviations.

^a^TG is not distributed normally are presented as medians with interquartile ranges.

### Correlation between repeated measurements of metabolic syndrome components at different times and ICC

ICCs were calculated to evaluate the correlation between repeatedly measured metabolic indicators. ICCs were 0.74 for BMI, 0.65 for HDL-c, and 0.60 for cMetS, showing a high level of correlation at the individual level. However, the level of correlation was low for the MAP and TG values and 0.27 for fasting blood glucose (Table 2).

**Table 2:** Correlation between repeated measurements of metabolic syndrome components at different times and ICC.

	Correlation coefficients								ICC
	3 years vs 5 years	5 years vs 7 years	7 years vs 8 years	8 years vs 9 years	9 years vs 10 years	10 years vs 11 years	11 years vs 12 years	12 years vs 13–15 years	3 years to 13–15 years
BMI (kg/m^2^)	0.546***	0.820***	0.898***	0.921***	0.952***	0.954***	0.913***	0.896***	0.739
MAP (mmHg)	0.109***	0.385***	0.531***	0.534***	0.273***	0.420***	0.302***	0.404***	0.353
TG (mg/dL)	0.207**	0.350***	0.445***	0.275***	0.494***	0.500***	0.620***	0.561***	0.368
FBG (mg/dL)	0.284***	0.231***	0.353***	0.311**	0.238**	0.461***	0.528***	0.475***	0.268
HDL-c (mg/dL)	0.556***	0.623***	0.685***	0.727***	0.748***	0.748***	0.809***	0.783***	0.648
cMetS	0.329***	0.523***	0.692***	0.666***	0.643***	0.717***	0.768***	0.724***	0.595

BMI, body mass index; MAP, mean arterial pressure; TG, triglyceride; FG, fasting blood glucose; HDL-c, high-density lipoprotein-cholesterol; ICC, Intra-class correlation, cMetS, continuous metabolic syndrome score.

* < 0.05, ** < 0.01, *** < 0.001.

### Trajectories of cMetS

**Figure 1:**
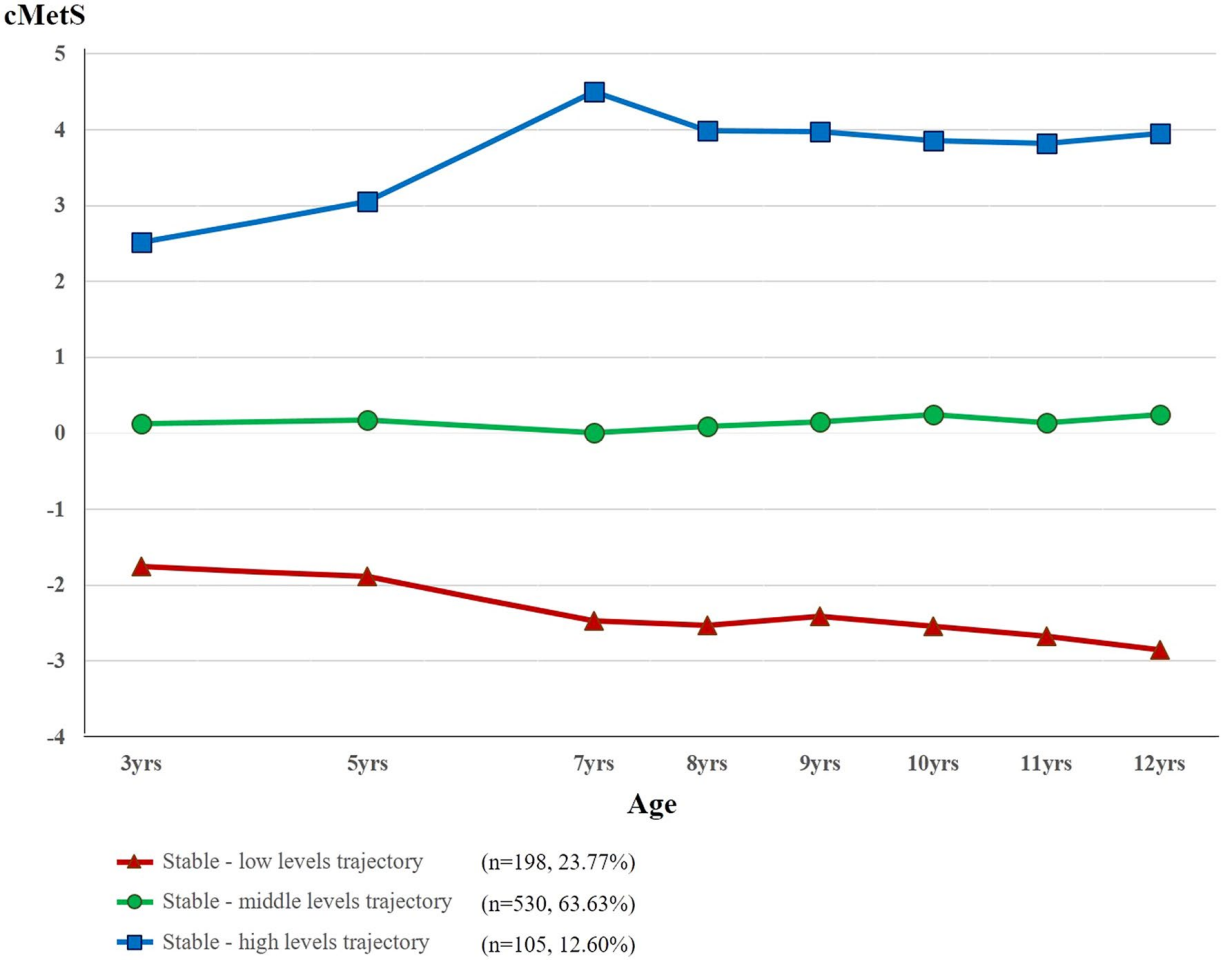
Trajectroies of cMetS from 3 to 12 years of age. *Abbreviation:* cMetS, continuous metabolic syndrome score.

Three cMetS trajectory groups were identified through trajectory analysis using the cMetS values measured from 3 to 12 years of age. Children with high cMetS at the age of 3 years had high cMetS throughout the observation period, and this stable trend was applied to children who had intermediate and low cMetS at the age of 3 years. The stable- middle cMetS trajectory group accounted for the largest proportion (63.63%) (Fig. 1). There was no difference in the basic characteristics according to the trajectory patterns, except for the mother’s education level (Supplementary Table II).

The average CIMT in the stable-high cMetS trajectory group was approximately 0.01 to 0.02 mm thicker than that of the other two trajectory groups, but the difference was not statistically significant (Table 3). Regarding the association between cMetS trajectory group and hs-CRP level, those in the stable-high group had a 4.50-fold greater risk of elevated hs-CRP than those in the stable-low group. This result remained significant even after adjusting for covariates. A significant gender difference in the association between trajectory group and hs-CRP was found, but not for CIMT. Moreover, when we analyzed the association between the trajectory group and hs-CRP by sex, the association was significant only in boys (Table 4).

**Table 3:** Mean comparison of CIMT between groups of cMetS trajectories.

				cMetS trajectory group						*p*
				Stable-low group (n = 47)		Stable-middle group (n = 129)		Stable-high group (n = 28)		
Crude model	CIMT-L (mm)	Mean ± SD		0.44 ± 0.07		0.45 ± 0.06		0.46 ± 0.07		0.23
	CIMT-R (mm)	Mean ± SD		0.44 ± 0.06		0.45 ± 0.07		0.46 ± 0.07		0.61
	CIMT-M (mm)	Mean ± SD		0.44 ± 0.05		0.45 ± 0.06		0.46 ± 0.05		0.29
Adjusted model 1^a^	CIMT-L (mm)	Lsmeans (95% CI)		0.44 (0.42,0.46)		0.46 (0.44,0.47)		0.46 (0.43,0.48)		0.23
	CIMT-R (mm)	Lsmeans (95% CI)		0.44 (0.43,0.46)		0.45 (0.44,0.47)		0.46 (0.43,0.48)		0.59
	CIMT-M (mm)	Lsmeans (95% CI)		0.44 (0.42,0.46)		0.45 (0.45,0.46)		0.46 (0.44,0.48)		0.28
Adjusted model 2^b^	CIMT-L (mm)	Lsmeans (95% CI)		0.43 (0.39,0.46)		0.45 (0.41,0.48)		0.44 (0.40,0.48)		0.24
	CIMT-R (mm)	Lsmeans (95% CI)		0.45 (0.41,0.48)		0.46 (0.42,0.49)		0.45 (0.41,0.50)		0.53
	CIMT-M (mm)	Lsmeans (95% CI)		0.44 (0.40,0.47)		0.45 (0.42,0.48)		0.45 (0.41,0.48)		0.27

SD, Standard deviation; LSMeans, least-squares means; cMetS, continuous metabolic syndrome score; CIMT-L, left carotid intima-media thickness; CIMT-R, right carotid intima-media thickness; CIMT-M, mean carotid intima-media thickness.

^a^Model1 was adjusted for sex.

^b^Model2 was adjusted for sex, age, monthly household income, parental history of disease (CVD, cardiovascular diease; DYS, dyslipidemia; HTN, hypertension; DM, diabetes mellitus.), weekly vigorous-intensity physical activity, and secondhand smoking.

**Table 4:** Results of odds ratio for high hs-CRP risk according to cMetS trajectory groups.

cMetS trajectory group		hs-CRP					
		≤ 0.3 mg/dL	> 0.3 mg/dL	*p*	Crude model	Adjusted model 1^a^	Adjusted model 2^b^
		(n = 136)	(n = 59)		OR (95% CI)	OR (95% CI)	OR (95% CI)
Total	Stable-low group	34 (75.56%)	11 (24.44%)	0.00	Ref	Ref	Ref
	Stable-middle group	91 (73.98%)	32 (26.02%)		1.09 (0.49, 2.40)	1.10 (0.50, 2.42)	1.01 (0.43, 2.39)
	Stable-high group	11 (40.74%)	16 (59.26%)		4.50 (1.61, 12.54)	4.57 (1.63, 12.82)	5.57 (1.76, 17.56)
Boys^c^	Stable-low group	18 (81.82%)	4 (18.18%)	0.01	Ref		Ref
	Stable-middle group	39 (67.24%)	19 (32.76%)		2.19 (0.65, 7.38)		1.57 (0.43, 5.75)
	Stable-high group	4 (30.77%)	9 (69.23%)		10.13(2.04,50.16)		8.33 (1.53, 45.52)
Girls^c^	Stable-low group	16 (69.57%)	7 (30.43%)	0.06	Ref		Ref
	Stable-middle group	52 (80.00%)	13 (20.00%)		0.57 (0.20, 1.68)		0.38 (0.10, 1.45)
	Stable-high group	7 (50.00%)	7 (50.00%)		2.29 (0.58, 9.03)		3.2 (0.51, 20.94)

hs-CRP, High sensitivity C-reactive protein; OR, odds ratio; 95% CI, 95% confidence interval.

^a^Model 1 was adjusted for sex. *p* for interaction with sex (0.02).

^b^Model 2 was adjusted for sex, age, monthly household income, parental history of disease (CVD, cardiovascular diease; DYS, dyslipidemia; HTN, hypertension; DM, diabetes mellitus.), weekly vigorous-intensity physical activity, and secondhand smoking.* p* for interaction with sex (0.01).

^c^For the sex-stratified analysis, we adjusted for all covariates except sex.

In addition, the association between trajectory group and elevated hs-CRP risk was maintained in boys, even after excluding subjects with hs-CRP > 1 mg/dL (data not shown).

## Discussion

In this study, the cMetS trajectory during childhood (from 3 to 12 years of age) showed three distinct patterns, and cMetS levels were consistently maintained throughout the observation period in all three patterns. The children in the stable-high trajectory group accounted for 12.60% of the total subjects. In addition, hs-CRP was significantly higher in adolescents who belonged to the stable-high trajectory group during childhood compared to that in their counterparts, only in adolescent males.

The cMetS is a measure to assess the risk of MetS in children who do not have a comprehensive or accurate diagnostic standard; thus, cMetS can be a useful tool in pediatric research. There is no established, accurate diagnostic cut-off for metabolic syndrome in children^25^. Therefore, previous study performed a cluster analysis including triglycerides, BMI, HOMA, and SBP to identify a high-risk metabolic cluster. Subjects in the same cluster share similar traits, but are not similar to those in other clusters. In this analysis, we used cMetS score to assess the risk of MetS in children; this is considered a valid in pediatric research^26–28^. The validity of cMetS was evaluated in students aged 7 − 18 years through a national school-based surveillance program in Iran. This study showed that the average cMetS increased according to the number of MetS components included, and it was found that the discriminatory power for MetS prediction was good (area under the curve = 0.94)^15^. Furthermore, other studies in adolescents^29^ and children^27^ reported similar results.

Regarding the persistence of cMetS, several studies evaluated the correlation coefficient^30,31^, but no study has tried to identify trajectory patterns of cMetS using long-term follow-up data. In a previous study using the same data source, the trajectory of BP, a component of MetS, was analyzed using data from 65 subjects repeatedly measured from 3 to 10 years of age, and the results were similar to the results of this study^32^. The identified trajectory patterns showed that cMetS values were clearly distinguished by trajectory groups from the early period of life and remained stable during a long follow-up period from 3 to 12 years of age. This suggests that poor metabolic indicators in early life can be maintained even during childhood. Furthermore, in a study that analyzed 5803 people from 4 cohorts with an average follow-up of 22.3 years, childhood cMetS was associated with the incidence of adulthood MetS, and the risk of incident MetS was 2.14 times (95% CI 1.19–3.85) higher in men and 3.79 times (95% CI 1.89–7.77) higher in women per 1-SD increase in cMetS at 5 − 7 years of age^33^. In addition, it has been reported that metabolic abnormalities in childhood include an increase in type 2 diabetes and a high CIMT in adults. One study using 25-year follow-up data found that MetS in childhood (6 − 19 years old) predicted adult CVD^34^. Furthermore, a previous study demonstrated that children showing increased adiposity between birth and 14 years of age had the greatest insulin resistance at 14 years of age, and an increased risk of prehypertension or hypertension at 17 years^35,36^. Together with these studies, our findings support the evidence that interventions are needed early in life to improve poor metabolic health and prevent CVD later in life.

Because a long follow-up period is necessary to prospectively evaluate the effect of metabolic health in childhood on CVD in adulthood, surrogate markers of CVD risk are often used. In this study, we evaluated hs-CRP and CIMT as surrogate markers of CVD risk. Although there have been studies evaluating the relationship between hs-CRP and metabolic health^37–39^, in general, studies in children and adolescents are lacking. In addition, most of the studies were conducted using a cross-sectional design. In a previous study conducted by the authors of this study, a significant cross-sectional association between cMetS and hs-CRP at 13 − 15 years of age and BMI, one of the metabolic components, was found to be directly related to hs-CRP^16^. On the other hand, the results in the present study were obtained by prospective evaluation using a general population of children and showed that those who belonged to the stable-high cMetS trajectory group during childhood had a higher average hs-CRP in adolescence than those who belonged to other groups. In terms of critical periods, cMetS from the age of 7 years was found to be significantly related to hs-CRP in adolescence. Therefore, our results are meaningful in that we derived results for critical periods of disease susceptibility using repeated measured data from a long follow-up period. We found a significant gender difference in the association between trajectory group and hs-CRP. However, the confidence interval was large because the size of each trajectory group was small after stratifying by gender. Therefore, more research should examine this gender difference.

Atherosclerosis occurs mainly in middle-aged individuals, but it has been suggested that the development of vascular changes begins in early life^40^. Therefore, CIMT can be another surrogate marker of CVD risk as a marker of preclinical atherosclerosis and has been reported to be associated with CVD development^41^. A systematic review found that adiposity had a positive correlation with CIMT, but its significance was not observed in studies involving subjects younger than 12 years of age^42^. In cross-sectional studies, one study found that cMetS had a positive correlation with CIMT in children aged 6 − 11 years, but no correlation was observed in children aged 12 − 18 years^43^. Using data from five countries, another study of children aged 6 − 17 years found that in normal-weight children, those with at least one risk factor (high BP, TG, fasting glucose, and low HDL-c) had a 1.44 times (95% CI 1.03–2.02) higher risk for CIMT (≥ 90th percentile) than those without risk factors3^44^. As a prospectively evaluated study, the Cardiovascular Risk in Young Finns Study evaluated the relationship between the number of childhood risk factors (high LDL-c, SBP, BMI, and smoking) and the average CIMT in adulthood, and it was found that the mean CIMT tended to increase significantly depending on the number of risk factors at the age of 12 − 18 years, but this relationship was weak at the age of 3–9 years^40^. In addition, a previous study^33^ reported that high CIMT in adults was related to cMetS at 11 years of age. In this study, only cMetS at the age of 8 or 9 years was significantly associated with CIMT in adolescence, and the effect was not maintained thereafter. However, there is a limitation in comparing these results with the results of the present study because the evidence evaluated in a similar age group as that used in our study is insufficient. Therefore, further evaluations are required through follow-up studies.

There were several limitations to this study that should be considered when interpreting the results. First, the subjects were from a single hospital and did not necessarily represent all Koreans. Therefore, the generalizability of the results is limited. In addition, measurement errors in the indicators could have led to bias. However, our study also has an important strength; it identified longitudinal patterns of cMetS in childhood through trajectory analysis of repeated-measures data. We were also able to show that hsCRP, which is a surrogate marker of CVD risk, was significantly higher in adolescents whose cMetS was consistently high during childhood through longitudinal data analysis.

This study suggests that it is necessary to evaluate the level of metabolic indicators from early childhood to identify high-risk groups and provide early intervention to reduce the risk of cardiovascular disease later in life.

## Supplementary Information

Supplementary Tables.
